# Detection of sitting posture using hierarchical image composition and deep learning

**DOI:** 10.7717/peerj-cs.442

**Published:** 2021-03-23

**Authors:** Audrius Kulikajevas, Rytis Maskeliunas, Robertas Damaševičius

**Affiliations:** 1 Department of Multimedia Engineering, Kaunas University of Technology, Kaunas, Lithuania; 2 Department of Applied Informatics, Vytautas Magnus University, Kaunas, Lithuania; 3 Faculty of Applied Mathematics, Silesian University of Technology, Gliwice, Poland

**Keywords:** Posture detection, Computer vision, Deep learning, Artificial neural network, Depth sensors, Sitting posture, e-Health

## Abstract

Human posture detection allows the capture of the kinematic parameters of the human body, which is important for many applications, such as assisted living, healthcare, physical exercising and rehabilitation. This task can greatly benefit from recent development in deep learning and computer vision. In this paper, we propose a novel deep recurrent hierarchical network (DRHN) model based on *MobileNetV2* that allows for greater flexibility by reducing or eliminating posture detection problems related to a limited visibility human torso in the frame, i.e., the occlusion problem. The DRHN network accepts the RGB-Depth frame sequences and produces a representation of semantically related posture states. We achieved 91.47% accuracy at 10 fps rate for sitting posture recognition.

## Introduction

Machine learning and deep learning has shown very good results when applied to various computer vision applications such as detection of plant diseases in agriculture (Kamilaris & Prenafeta-Boldú, 2018), fault diagnosis in industrial engineering (Wen et al., 2018), brain tumor recognition from MR images (Chen et al., 2018a), segmentation of endoscopic images for gastric cancer (Hirasawa et al., 2018), or skin lesion recognition (Li & Shen, 2018) and even autonomous vehicles (Alam et al., 2019).

As our daily life increasingly depends on sitting work and the opportunities for physical exercising (in the context of COVID-19 pandemic associated restrictions and lockdowns are diminished), many people are facing various medical conditions directly related to such sedentary lifestyles. One of the frequently mentioned problems is back pain, with bad sitting posture being one of the compounding factors to this problem (Grandjean & Hünting, 1977; Sharma & Majumdar, 2009). Inadequate postures adopted by office workers are one of the most significant risk factors of work-related musculoskeletal disorders. The direct consequence may be back pain, while indirectly it has been associated with cervical disease, myopia, cardiovascular diseases and premature mortality (Cagnie et al., 2006). One study (Alberdi et al., 2018) has demonstrated that body posture is one of the best predictors of stress and mental workload levels. Another study linked postural instability and gait difficulty with a rapid rate of Parkinson’s disease progression (Jankovic et al., 1990). Posture recognition is also relevant for disabled people (Ma et al., 2020) and elderly people for proper health diagnostics (Chen et al., 2018b) as the sedentary behavior has a negative effect on people’s wellbeing and health. Therefore, the solutions that would improve the daily living conditions of both healthy and spine pain affected people in the context of assisted living environments (Maskeliunas, Damaševičius & Segal, 2019).

While there are existing classical approaches for human posture prediction, unfortunately, they generally assert that entire human skeleton is visible in frame. Even though those assumptions of scene composition are valid, with everyone moving to their home offices, meeting them is simply not feasible. Not everyone is capable of having complex multi-camera setups to track their body posture. For this reason, there is a need for a solution that is able to inform the end-user (or their care provider) of their bad posture with cheaply available consumer sensors in order to improve their wellbeing without real-time supervision. With the renaissance of machine learning, and its application in computer vision tasks, we are able to solve a lot of complex tasks using black box models by shifting the majority of computations from the end-user device into the training stage. For this reason, artificial neural networks have been used in wide variety of applications. In this article, we propose a novel deep recurrent hierarchical neural network approach for tracking human posture in home office environments, where majority of the person sitting at the desk and therefore is occluded from the camera. Additionally, a pilot of 11 test subjects is made in order to validate our approach effectiveness.

## Related work

The existing solutions such as orthopedic posture braces may not be viable solution due to other underlying medical conditions. Computer-aided posture tracking combined with behaviour improvement techniques due to continuous monitoring and self-assessment can contribute to remedy this issue (Dias & Cunha, 2018). The most prominent solution to this problem is skeleton based posture recognition (Jiang et al., 2015) using commercially available depth sensors such as *Microsoft Kinect* (Zhang, 2012) and *Intel Realsense* (Keselman et al., 2017). However, these solutions generally depend on some assertions, i.e., camera calibration settings, lightning conditions, expected posture (Hondori & Khademi, 2014), often making the results unreliable.

For identifying inadequate posture wearable textile sensors (García Patiño, Khoshnam & Menon, 2020), inertial and magnetic sensors attached to human body (Bouvier et al., 2015), depth cameras (Ho et al., 2016), radio-frequency identification tags (Saab & Msheik, 2016), 3D motion analysis (Perusquía-Hernández et al., 2017), video surveillance (Afza et al., 2021), Kinect sensors (Ryselis et al., 2020) and sensors attached to office chairs (Zemp et al., 2016; Bibbo et al., 2019) were used, while registering body posture parameters such as forward inclination, distance to the computer, and relative skeletal angles. However, the camera-based systems have demanding requirements for distance, proper lighting, calibration and non-occlusion.

Another approach focuses on wiring sensors directly to the human body to acquire data, although it limits the freedom of movement for work activities (Arnold et al., 2020). Despite these achievements, it is still quite difficult to recognize posture in real-time or correctly identify transitional activities in real-world environments (Nweke et al., 2019) as the recognition of fine-grained activities such as correct or incorrect cases of sitting postures is still a problem (Chin et al., 2019).

Currently, the state of the art in non-invasive posture tracking is depth and image processing (Abobakr, Hossny & Nahavandi, 2018; Matthew et al., 2019; Camalan et al., 2018). For example, Huang & Gao (2019) reconstruct realistic 3D human poses using the 3D coordinates of joint points captured by the depth camera and employ conformal geometric algebra to improve human limb modelling. Li, Bai & Han (2020) used OptiTrack and Kinect v2 to get and transfer data into a human skeleton coordinates. They used random forest regression learn the joint offset regression function, and adjust the skeleton based on the predictions on joint offset. Finally, as a result, they can determine the motions based on predicted posture. Liu et al. (2020) suggested 3D Convolutional Neural Network (CNN), called 3D PostureNet, while Gaussian voxel modeling is adopted to represent posture configurations. The method allows to eliminate the coordinate deviations caused by various recording environments and posture displacements. Pham et al. (2019) exploit Deep CNNs based on the DenseNet model to learn directly an end-to-end mapping between the input skeleton sequences and their action label for human activity recognition. The network learns spatio—temporal patterns of skeletal movements and their discriminative features for further downstream classification tasks. Sengupta et al. (2020) detect skeletal joints using mmWave radar reflection signals. First, the reflected radar point cloud. Next, CNN was trained on radar-to-image representation and used to predict the position of the skeletal joints in 3-D space. The method was evaluated in a single person scenario using four primary motions. The method has shown to be robust in adverse weather conditions and deviations in light conditions.

However, none of the above mentioned methods are applicable when only the upper part of the body is visible. Some methods tried to tackle this problem by exploiting the temporal relationship between the body parts to deal with the occlusion problem and to get the occluded depth information (Huang, Hsu & Huang, 2013) or by recreating a topological character (Bei et al., 2017), yet they still require a recreation of a full body skeleton.

To address this problem, we propose a novel approach for human posture classification by using a supervised hierarchical neural network (Liu et al., 2019) that uses the RGB-Depth data as input. Our method extends *MobileNetV2* (Sandler et al., 2018) neural network to include the recurrent layers. This allows the network to learn from a sequence of images by adding the dimension of time to our feature list. Allowing the network to use the context of what happened in previous frames to make predictions. This is an improvement over existing methods for skeleton prediction as this allows for our approach to predict user posture in more complex environments, for example, when a person is sitting in front of an office desk thus a large portion of his/her body is occluded. Such position would cause other known skeleton-based posture prediction methods to fail, due to lack of data provided by the sensors to infer the full human skeleton.

Our novelty is the use only a simple depth camera, so the subject does not need to wear any sensors on their body nor have entire body visible in sensor field of view. In fact, only the upper 30% of the body may be visible, whereas when using the Kinect style sensor, the lower legs must be visible or generated artificially to allow the reconstruction of the skeleton or, otherwise, the recognition process fails. Our approach does not rely on a (visual or artificial) reconstruction of the full skeleton and, thus, allows for the detection of posture in advanced scenarios such as sitting at a desk, where a camera often receives very limited information.

## Methods

### Network architecture

Our preliminary analysis has shown that it is very hard to predict human posture based on a single shot. For this reason, we opted to use time sequences with *n* = 4 frames. However, during training the input has a variable length of 1 *≤ n ≤* 4, with each frame being about a second apart to reduce the dependence on the previous frames.

We selected to use deep convolutional recurrent neural networks for they have shown some of the best capabilities when it comes to similar tasks requiring to predict sequences of data as with natural speech recognition (Sundermeyer, Ney & Schlu, 2015; Graves, Mohamed & Hinton, 2013) or even traffic prediction (Ji & Hong, 2019).

**Figure 1:**
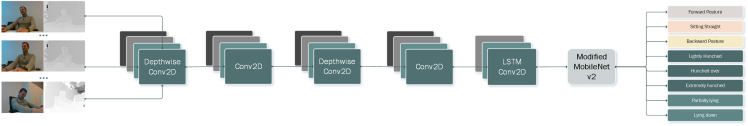
Our recurrent hierarchical ANN architecture using *MobileNetV2* as the main backbone. It takes the RGB-D frame sequence as input and outputs the flattened prediction tree as a result.

**Figure 2:**
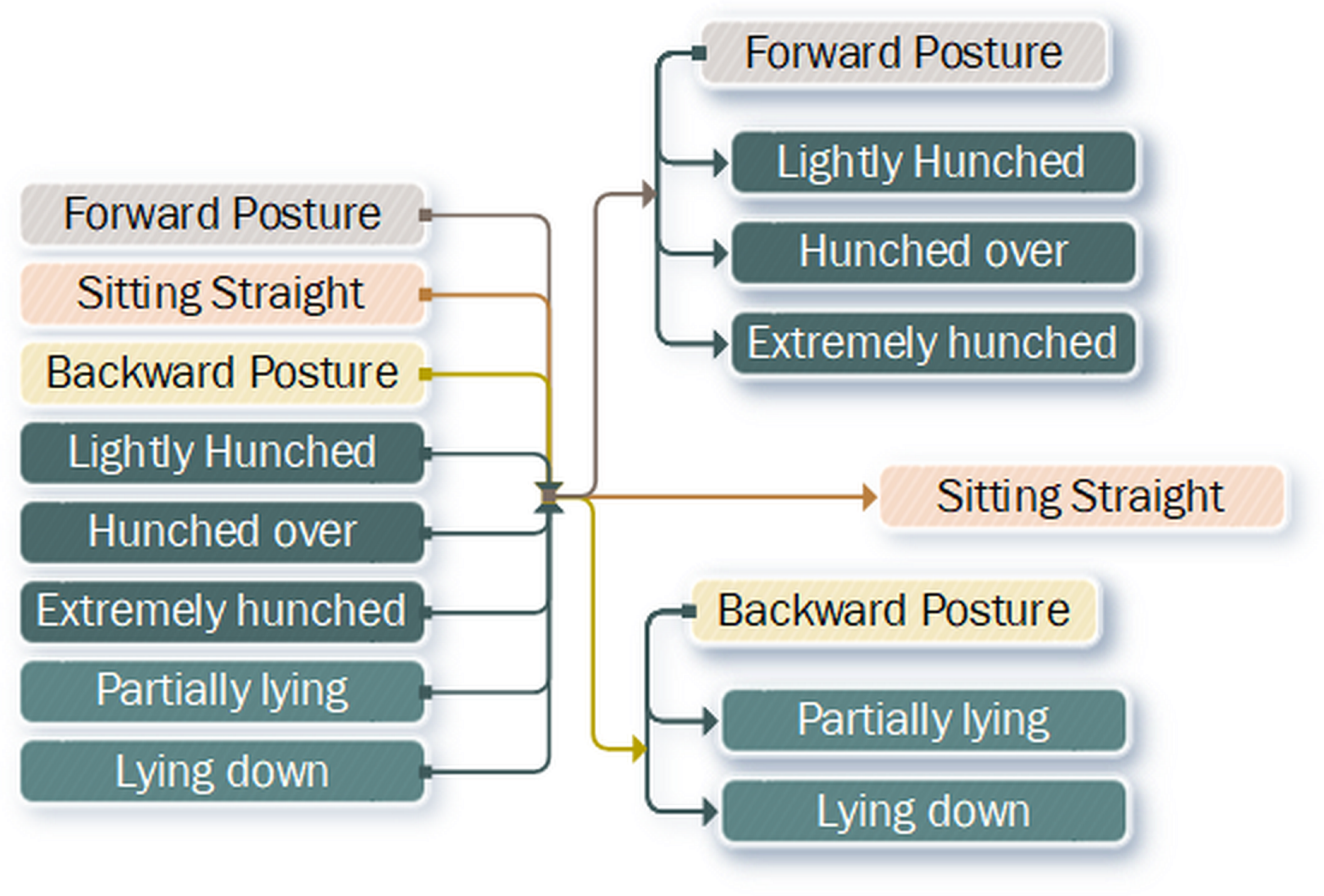
Flattened hierarchy representation of postures expanded into a hierarchical tree.

The input of our network architecture (Fig. 1) is the RGB-D frame sequence that is fed into depth-wise convolutional block (Zhang et al., 2019), which reduces the dimensionality of each frame by a factor of two, without losing each individual channel’s influence on the output. This is due to depth-wise convolutions applying separate kernels for each channel. This is then followed by a convolutional layer in order to extract the best individual features, which is followed by a second dimensionality reduction layer. We do this because our input frames are captured at 640 × 480 px resolution, which is the maximum hardware resolution of the *Intel Realsense D435i* device. Reducing the dimensionality twice leaves us with 128 features, each of 160 × 120 px resolution. At this stage, we use Long Short Term Memory (LSTM) convolutional block (Xu et al., 2020; Li et al., 2020), which is tasked to extract 32 most useful features in the entire sequence. For our main neural network backbone, we use *MobileNetV2*, which is the extension of *MobileNet*, for it has show to achieve great results in predictive capabilities (Howard et al., 2017; Zhou et al., 2019), however, the architecture itself is relatively light-weight for it is designed to be used in low power devices such as mobile devices, unlike for example, *YOLOV3*, which while having impressive recall results (Redmon & Farhadi, 2018), is much more complex and has a substantially poorer performance. The *MobileNetV2* output is then connected to a global average pooling layer in order to reduce dimensionality and improve generalization rate (Zhou et al., 2016). Finally, the output is subsequently connected to a fully-connected layer, which represents the flattened representation of posture state prediction hierarchy, which can be seen in Fig. 2.

Our entire ANN setup can be seen in Table 1. After each of two bottleneck layers we additionally use spatial dropout layers as it is shown to improve generalization during training and reduce the effect of nearby pixels being strongly correlated within the feature maps (Murugan & Durairaj, 2017), each with dropout probability of 0.2, whereas the spatial dropout post LSTM cell has a dropout probability of 0.3, because the higher the network is upstream the more dropout layers influence the entire network, therefore high values upstream may cause the network to be more unstable and difficult to train. The dropout layer before the output layer however, has a dropout probability of 50%. Aggressive dropout values reduce the chance that the model is will overfit by training on noise instead of image features. All our previous layers up to this point had used Rectified Linear Unit as our activation function in order to impose non-linearity into our model for it has shown better results and improved performance due to its mathematical simplicity in CNNs (Hanin, 2019). However, for the last layer we opted to use the *sigmoid* activation due to our network outputting hierarchical values and acting as multi-label classifier, while the *softmax* activation is more useful for multi-class classification tasks.

**Table 1:** Layers of the proposed neural network architecture for human posture recognition.

Type	Filters	Size	Output
Input	–	–	*t* × 640 × 480
Depthwise convolution	–	11 × 11/2	*t* × 320 × 240
Convolution	64	1 × 1	*t* × 320 × 240
Spatial dropout *P*(*x*) = 0.2	–		*t* × 320 × 240
Depthwise convolution	–	5 × 5/2	*t* × 160 × 120
Convolution	128	1 × 1	*t* × 160 × 120
Spatial dropout *P*(*x*) = 0.2	–		*t* × 160 × 120
LSTM convolution	16	3 × 3	160 × 120
Spatial dropout *P*(*x*) = 0.3	–		160 × 120
*MobileNetV2*	–	–	4 × 5
Global average pooling	–	–	1,280
Dropout *P*(*x*) = 0.5	–		1,280
Fully-connected (sigmoid)	–	–	8

### Algorithm of sitting posture detection

**Figure 3:**
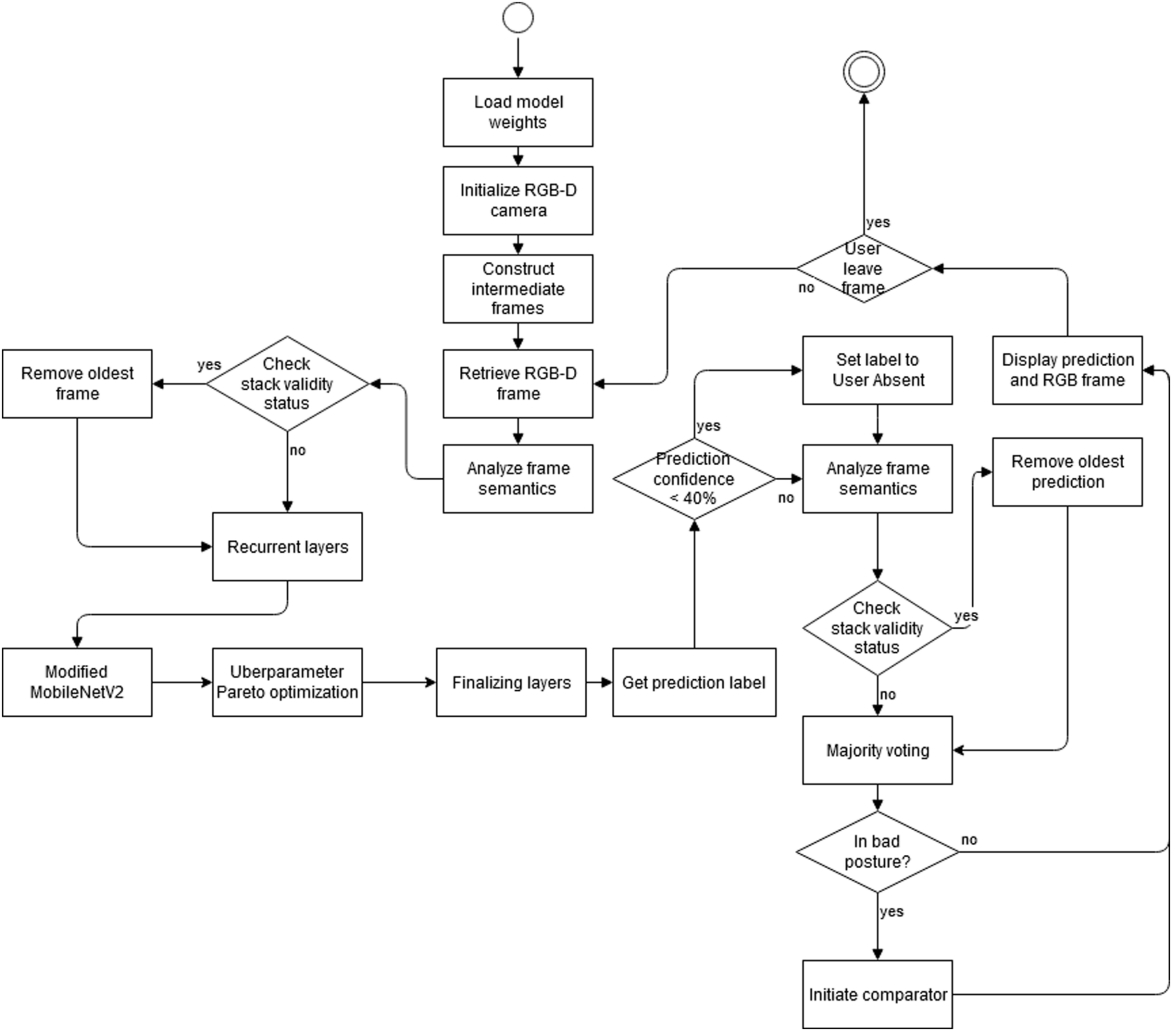
Activity diagram of the proposed method for sitting posture state recognition.

Figure 3 depicts algorithm of our enhanced posture detection solution. Process starts with the initialization of model weights for sorting out the RGB-D camera output (both depth and RGB as varying on the condition either modality can provide compensation features). Algorithm then tries to reconstruct intermediate frame for retrieval and analysis of frame semantics, which are then used for stack validity status verification. Assuming the condition, analysis starts in the recurrent layers of our modified MobileNet v2 architecture, with Pareto-Optimal berparameter optimization (Plonis et al., 2020). The model then assigns prediction labels and algorithm further tries to improve the quality by firing smart semantic prediction analyzer, checking not only the output value but probable output status for a combined confidence level of <40%, as an improved determinator for further frame semantic analysis. A final validity status is then initiated, depending on condition leading to a majority voting and a very reliable detection of bad posture. Algorithm was designed to work continuously and is able to automatically stop processing to stay compatible with green computing paradigm (Okewu et al., 2017) to save energy.

### Prediction of posture states

We adopted the semantic matchmaking approach (Ruta et al., 2014) to describe the semantic relationship between different postures using an ontological tree for analysis, reasoning and knowledge discovery. In order to extract the specific prediction label we parse the posture hierarchy tree (Fig. 2), first, by checking, which posture state is most likely according to the neural network. Once we know, which posture type is most likely to be represented in the frame sequence, we proceed to the sub-nodes and check their predictions. We continue this search until we find the leaf node, which represents the actual label. This approach allows us to filter out the most likely path that is seen in the frames. This is helpful in cases when the similarities between postures is large. For example, all forward postures share the same characteristics—shoulders are not at 90 degree angle, and the head is positioned forward with respect to the body. This allows us to ignore all the weight influences, where for example, the person is lying down. Additionally, the further down the tree the label leaves are the less of an overall recall error is, due to each level of the tree being ontologically similar, for example, predicting *lying down*, instead of *partially lying down* is a smaller error than predicting *hunched over*.

#### Loss function

One of the reasons why we use the flattened final layer to represent our posture hierarchy is because we can represent our problem as multi-label classification (Wang et al., 2019). This allows us to use binary cross-entropy (Eq. (1)) in order to calculate the loss between expected output and ground truth:

(1) $${H_p} = \sum\limits_{i = 1}^N {\hat y_i} \cdot log({y_i}) + (1 - {\hat y_i}) \cdot log(1 - {y_i})$$

Binary cross-entropy classifier is fit for our multi-label classification task (Wu et al., 2018) as each of our cells output is a binary one and more than one cell can be positive at a time, depending on how deep the classification is, as opposed to the categorical cross-entropy, which is a solution for multi-class tasks, where the input can yield only a single-class output.

### Network training

For training a neural network various optimization methods have been proposed. However, one of the most popular optimization methods due to its computational efficiency allowing training ANN on large datasets more easily on weaker hardware in addition to the ability to achieve faster convergence than other methods is Adam (Kingma & Ba, 2015). For these reasons, we had opted to use Adam for training using the initial training rate of 5*e*^−4^, with a batch size of *eight*.

Additionally, we perform data augmentation as it has shown to improve ANN generalization (Fawzi et al., 2016). We perform horizontal image flipping in order to increase the view count, and perform random hue and saturation changes, aiming to increase stability against different lightning conditions as all of our video sequence instances were recorded during same time frame, therefore, maintaining nearly identical lightning. In all cases, the identical augmentation values are used for all images in the same series with the same probability of performing image flipping, hue and saturation augmentation being 50% independently. Random hue shift is performed in the range of *h* = [0, 2*π*) radians, while the saturation has the random range of *s* = [0, 2].

### Data collection

ANNs have the benefit of doing all the heavy work upfront during the training therefore, allowing to improve system runtime by reducing the number of required calculations (Holden et al., 2019). However, this approach depends on the quality of the training data, which can be defined in terms of the size of available samples, class balance and even the correctness of the labels. Our approach depends on both color and depth information. Unfortunately, still there are no publicly available labeled human posture dataset that additionally provides depth information. For our experiments, we have devised a methodology to create such dataset. The data collection procedure consists of *two* stages.

#### Stage I

The person starts by sitting up straight. This position is then filmed for 30 s. Afterwards, the person is instructed to lightly hunch forward, which is followed by another 30 s of sitting in this position. Afterwards, the person is again instructed to hunch more, emulating their average hunching posture. After the filming, the subject is instructed once more to hunch forward in order to emulate the worst possible forward posture. Once the 30 s have been recorded in this posture, the person is then instructed to sit up straight for an additional 30 s to get used to this position. Then, we start emulating the bad backwards posture, i.e., lying down in the chair. The person is instructed to partially lie in the chair for 30 s, after which he/she is instructed to do it twice more in increasingly bad posture positions giving us three sets of bad forward and backward posture examples.

#### Stage II

The person is instructed to initially sit up straight. Then the person is instructed to start slowly counting from *one* to *five*, while slowly worsening their forward posture. When the person finishes counting, he/she is expected to be in the worst forward posture they imagine. Afterwards, the subject returns to straight position. This action is repeated for *five* times. Once the forward posture data is recorded, the person is asked to perform the similar action, this time with backwards posture, where once again when they finish counting, they are fully lying in the chair.

Each of the stages is recorded three separate times using different camera perspectives at *10 o’clock, 12 o’clock and 3 o’clock*. The person is filmed in front of the computer desk and during the filming they are asked to interact with the table in their current posture how they imagine they would sit on the table. This can range from drawing on a piece of article, to checking the drawers, using keyboard or even holding their head with their hand.

When collecting our dataset, we asked 11 subjects (seven men and four women) to perform the posture emulation tasks. The informed consent was obtained, while we followed strictly the requirements of the Helsinki declaration. The research was approved by the Institutional Review Board, Faculty of Informatics, Kaunas University of Technology (2018-09-24 No. IFEP201809-2). Further expansion of dataset to include different body types or disabilities may additionally improve the results in more real world cases.

### Data labelling

Once the data is collected it must be labeled manually. However, one of the issues when labeling data we have noticed that has caused some of the data points to be thrown out completely is for a person to actually differentiate properly what posture that person is in. Even though the filming took in relatively discrete time intervals, some subjects may take longer/shorter to perform specified actions, they may attempt to *fix* their posture due to it being uncomfortable for them, etc. Additionally, some people have indiscernible *sitting straight* and *lightly hunched* posture, as their normal posture is already biased towards leaning head forward. Therefore, the labeling of such data is a challenge due to its subjectiveness as bad data labels may poison the network and cause it to overfit instead of generalizing.

Using our recorded dataset, we have extracted these labels: *sitting straight*, *lightly hunched*, *hunched over*, *extremely hunched*, *partially lying* and *lying down*. While we have three backwards posture angles, we opted for only two backwards posture labels as it is difficult to objectively measure *lying down* and *extremely lying down* as in multiple cases subjects barely made any movements.

### Dataset

Our dataset consists of 66 different captured video sequence instances totaling 133 min of recording, which we split into individual labeled frames. We used 10-fold cross-validation. For training, we had split the data from each individual in 90:10 ratio instead of splitting the frames, as this gives more objective results, because similar frames from the same captured video will not be a part of evaluation, thus artificially increasing the recall rate. We can see the number of frames in training and testing frames in Table 2, additionally we can see that dataset is slightly skewed towards *sitting straight* and *lying down* due to the dataset being not completely balanced. While class imbalance may cause issues in generalization of the network, we believe that the imbalance is not high enough to have a noticeable impact. Finally, the examples of images in the dataset are given in Table 3 (right side view). The subjects presented in the images have provided their informed consent for publication of identifying images in an online open-access publication.

**Table 2:** Frame count in the dataset.

Posture class	Training	Testing	Dataset (%)
Sitting straight	3390	505	21.53
Lightly hunched	2230	200	14.16
Hunched over	2534	321	16.09
Extremely hunched	1918	182	12.18
Partially lying	2053	339	13.04
Lying down	3622	302	23.00

**Table 3:** Examples of images in dataset (right side view).

Posture class	RGB and Depth images
Sitting straight	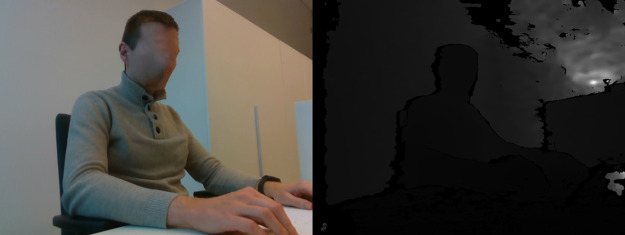
Lightly hunched forward	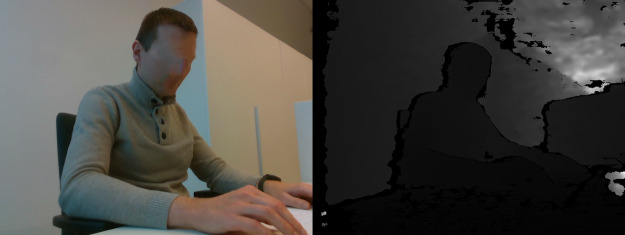
Hunched over forward	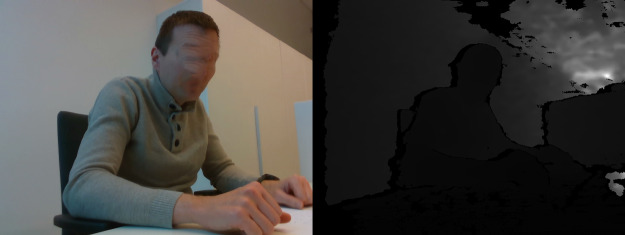
Extremely hunched forward	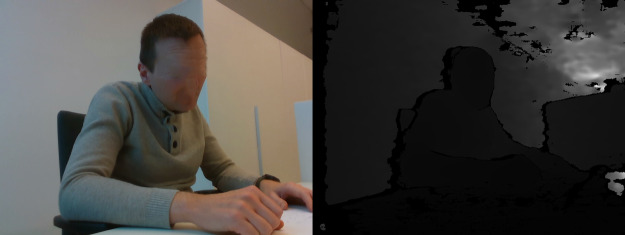
Partially lying down in the chair	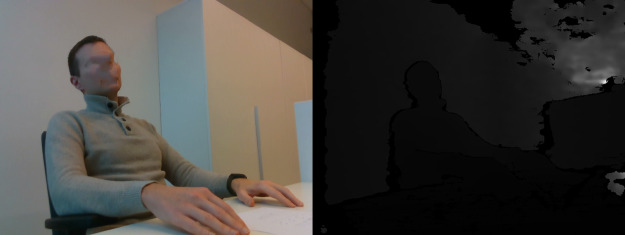
Lying down in the chair	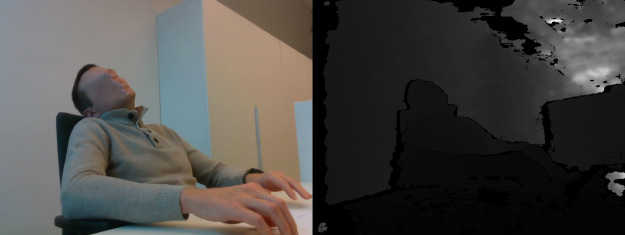

## Results

### Accuracy

**Figure 4:**
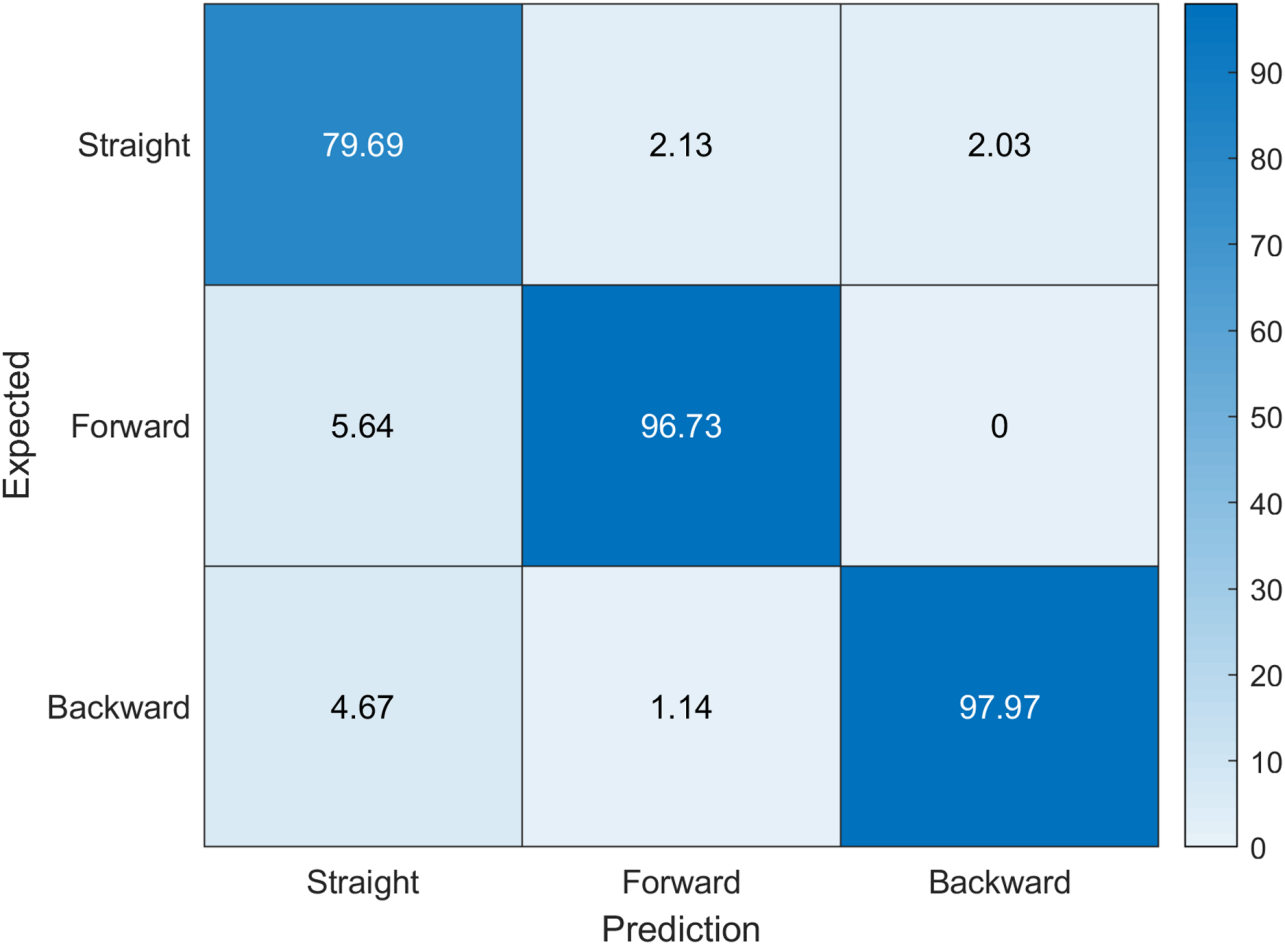
Confusion matrix indicating expected labels versus network predictions. Accuracy values are given in percents. Diagonal values indicate correct predictions.

We evaluate the prediction correctness against ground truth in two stages: using final prediction labels (*sitting straight, lightly hunched, hunched over, extremely hunched, partially lying, lying down*); and intermediate branch predictions (*forward posture, backward posture, straight posture*). This provides us better insight on the prediction results as this will show both absolute error and intermediate branch error. The confusion matrix for the first case can be seen in Fig. 4. In the first case, we achieved an accuracy of 68.33%, sensitivity of 0.6794, specificity of 0.9372 and *f*-score of 0.6789. Note that the network has achieved a high specificity rate, which means that it can effectively recognize the subjects, who do not have the posture problems. As we can see from the confusion matrix (Fig. 4), the biggest issues arise in prediction regarding *hunched over* and *extremely hunched* labels. The proposed network model had a hard time discerning between these two values. This indicates that either our dataset for these two labels have little variation and the positions are very similar, or that one of the labels has been mislabeled and has poisoned the predicted values. This suggests that further investigation in our dataset is definitely needed.

However, all largest misclassification values occur between neighbouring classes (*extremely hunched* vs *hunched over*—49.5%), (*hunched over* vs *extremely hunched*—40.66%), (*partially lying* vs *lying down*—28.15%), (*lying down* vs *partially lying*—19.47%), suggesting that perhaps the need for some fuzzification of class definitions and interpretation of results, or that these posture classes should be combined.

**Figure 5:**
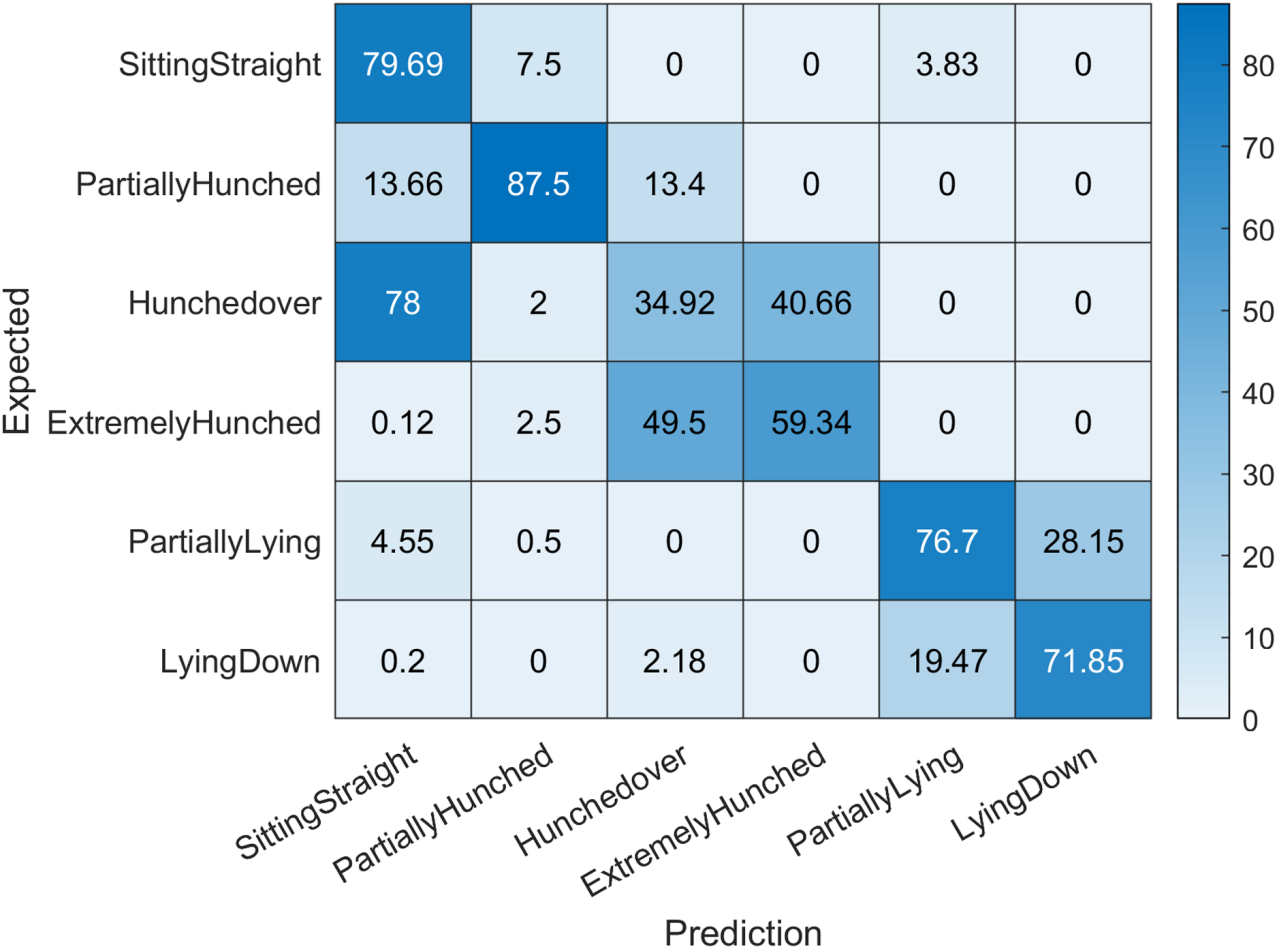
Confusion matrix indicating bottom level expected labels versus network predictions. Accuracy values are given in percents. Diagonal values indicate correct predictions.

Our dataset depends on the expert interpretation of what they are seeing in the camera, which may be the cause of this disparity. Performing data labeling by more experts may improve the results as this would reduce the ambiguity in our dataset that we have due to a limited number of experts labeling the data. However, the network is accurate enough that it can suggest the labels in further labeling processing. This would change our solution from being supervised machine learning into semi-supervised or even completely unsupervised machine learning approach. Notwithstanding, this is beyond the scope of our research. However, if we investigate further we can see from Fig. 5 that the root posture prediction has better results, where the network model manages to generalize between *forward posture*, *backward posture* and *straight posture* cases.

This partial confusion matrix (Fig. 5) makes it clear that while some finer detail in our dataset is less objective and is difficult for the network to generalize, the neural network itself is adept in solving the classification of the base postures with the mean accuracy rate of 91.47% (sensitivity 0.9185, specificity 0.9595, *f*-score 0.9132 and kappa 0.8081). The bottom level (root) labels are more than enough in a lot of cases when it comes to posture recognition tasks that do not require precise user angle extraction. Additionally, when comparing partial and full confusion matrices we can see that the deeper levels additionally have lower false negative results, indicating the addition of the hierarchical structure for prediction can inherently improve the prediction results in deeper levels due to the semantic connections between labels.

### Performance

Due to fact that our approach uses *MobileNetV2* as the backbone for our ANN that means that is lightweight and can be used in real-time applications. Our method performs a posture prediction on average in 94 ms (which corresponds to 10 fps rate) on a workstation with the following specifications: *Intel i7-4790* CPU with 16GB of RAM, *nVidia 1070* GPU with 8GB of GDDR5 VRAM.

### Comparison

We compare our results with the results of other authors in Table 4.

**Table 4:** Comparison of posture recognition methods.

Method	Frame resolution, px	Frame rate, fps	Accuracy, %	Task	Reference
Real-time deformable detector	320 × 240	10	75.33	Hand posture recognition	Hernandez-Belmonte & Ayala-Ramirez (2016)
Ensemble of InceptionResNetV2	640 × 480	n/a	95.34	Four postures (standing, sitting, lying, and lying crouched)	Byeon et al. (2020)
LVQ (learning vector quantization) neural network	640 × 480	333	99.01	Five full-skeleton postures (standing, sitting, stooping, kneeling, and lying)	Wang et al. (2016)
Multi-stage convolutional neural network (M-CNN)	n/a	5	98.70	Two postures for fall detection	Zhang, Wu & Wang (2020)
LVQ neural network	48 × 16	10	99.95	Eight postures (stand, hand raise, akimbo, open wide arms, squat, toe touch, crawl, and lie)	Gochoo et al. (2018)
Deep CNN	24 × 8	9	99.99	26 yoga postures	Gochoo et al. (2019)
D CNN	n/a	n/a	98.16	Detection of 10 standstill body poses.	Liu et al. (2020)
Deep recurrent hierarchical network	640 × 480	10	91.47	Spine posture recognition while sitting	This paper

**Note:**

n/a, data is not available.

Our method allows to achieve the real time sitting posture recognition with the same or better recognition accuracy and video resolution than other similar state-of-the-art methods. For example, Wang et al. (2016) achieved higher accuracy and recognition rate, however their method require the visibility of full skeleton detected by Kinect sensors and not occluded by any obstacles. Gochoo et al. (2018, 2019) achieved a very high recognition rate using three low resolution thermal sensors placed around the subject to recognize eight postures and 26 yoga postures, respectively, but no occlusions were allowed either. Tariq et al. (2019) used Kinect and additional motion sensors from smartwatch to achieve the required level of accuracy.

## Discussion

The training of neural network depends on hardware used for recording. We used *Intel Realsense D435i*, but the results may be worse when using different hardware, for example, *KinectV2* as these two devices produce different noise in their depth fields. This may cause the network to have poorer results when compared to the one that it has been trained on. However, we are not able to validate this claim. Additionally, when testing the network using the real-time camera feed we had noticed that while relatively similar and their mirror image angles work it may have lower precision rates with something more extreme like placing sensor very high or very low relative to the table or user.

Finally, when using in real world application, one of the measures to improve prediction stability is to use majority voting on the preceding 10 video frames. This is performed by taking the prediction label that had appeared the most times in the previous recorded 10 frames. This technique can improve the stability of the predictions as a single video frame will no longer change the prediction results. However, the predictions will have a delay, due to previous video frames influencing the result for a short period of time.

Another limitation of this study is a small number of subjects (11), all healthy, which may have influenced the validity of the results. The age range and gender diversity of the subject group was limited. In future, we will have to extend the subject group to include various professional/occupational groups as well as school children and adolescents as well as people with different body types and disabilities in order to improve the results for real world cases.

## Conclusion

We have proposed an extension of the *MobileNetV2* neural network, which allows the use of sequential video data as an input, therefore, allowing for the deep neural network to extract important temporal features from video frames, which would otherwise be lost when compared to a single-frame classification while still being capable of the single-frame prediction due to being biased towards the last frame. We have improved the top-layer of the *MobileNetV2* architecture by adding the hierarchical data representation, which acts as a semantic lock for top-level label classification by filtering out the invalid class labels early. Additionally, we have performed a pilot study based in which we suggest the methodology required to collect the training dataset and validation datasets. Further improvements in dataset collection methodology can be made in order to account for different body shapes, disabilities and removing labeling ambiguities. The proposed posture classification approach is highly extensible due to its flattened tree representation, which can be easily adapted to the already existing posture classification tasks with the depth of the ontological semantic posture model being one of the driving factors for classification quality. Based on our validation data giving us a classification accuracy of 91.47% in predicting three main sitting posture classes (*backward posture*, *forward posture* and *straight posture*) at a rate of 10 fps. Finally, unlike in related work, our method does not depend on the skeletal predictors, therefore we can perform the sitting human posture prediction when only as low as 30% of the human torso is visible in the frame. For these reasons, we believe that our approach is more robust for real-time human posture classification tasks in the real-world office environment.

## Additional Information and Declarations

### Data Availability

The following information was supplied regarding data availability: Data and code are available on GitHub: https://github.com/realratchet/SitStraightNet
